# Incomplete footprint coverage under tension in repair of isolated supraspinatus full-thickness tear

**DOI:** 10.1038/s41598-021-86800-3

**Published:** 2021-04-01

**Authors:** Tae-Hwan Yoon, Sung-Jae Kim, Yun-Rak Choi, Du-Seong Kim, Yong-Min Chun

**Affiliations:** Department of Orthopaedic Surgery, Arthroscopy and Joint Research Institute, Severance Hospital, Yonsei University College of Medicine, 50-1 Yonsei-ro, Seodaemun-gu, CPO Box 8044, Seoul, 03722 Korea

**Keywords:** Tendons, Pain

## Abstract

Although it is well known that repairing large or massive tears under tension may have an adverse effect on healing of the repaired tendons, only few studies have addressed this issue in medium-sized isolated supraspinatus full-thickness tear. The purpose of this study was to compare the clinical outcomes and structural integrity of arthroscopic rotator cuff repair with tension versus without it. This study retrospectively investigated 90 patients who underwent arthroscopic repair in a single-row for medium-sized isolated supraspinatus full-thickness tear. The patients were assigned to either repaired under tension (Group A, n = 38) or repaired without tension (Group B, n = 52) groups. Functional outcomes were assessed using the patient reported subjective values and the active range of motion (ROM). Postoperative radiographic evaluation was performed 6 months after the surgery to assess the structural integrity of the repaired tendons. Changes in the subjective shoulder scores from initial to 2 years after surgery showed no statistical significance between the two groups. The ROMs measured at initial and 2 years after surgery also showed no statistical difference between the two groups. Postoperative radiological evaluations found a significantly higher re-tear rate in Group A (28.9%, 11/38) than in Group B (9.6%, 5/52). The torn cuff tendons that were repaired under tension as retraction with limited mobility had significantly higher re-tear rate despite having immobilized for 6 weeks after surgery, but their clinical outcomes showed no significant difference from the outcomes of repaired tendons without tension.

## Introduction

It has been recommended to avoid excessive tension in arthroscopic rotator cuff repair^1–6^. However, despite improved surgical techniques to facilitate the cuff tendons’ mobility^7–9^, repairing the tears under tension is inevitable in large or massive rotator cuff tears and even in some medium-sized isolated supraspinatus full-thickness tears. Therefore, these operated shoulders should be immobilized in abduction to minimize undue tension or load at the repair site, maintaining the immobilization without early motion exercises, although delayed rehabilitation protocol is likely to induce postoperative stiffness^10–13^.

This postoperative stiffness has been known to cause severe postoperative pain, and it may become significant enough to develop sleep disturbance, especially in the early postoperative period. Although most re-tear cases are not related to poor clinical outcomes, nonetheless, the induced stiffness may be better than re-tear of the repaired rotator cuffs, considering the difficulty in revision surgery^14^.

Many studies have indicated the prognostic factors for reparability and postoperative structural integrity. In addition, these studies also examined a way to forecast postoperative clinical outcomes from preoperative magnetic resonance image (MRI) by analyzing the degree of muscle atrophy/fatty infiltration, involvement of the infraspinatus tendon, and tear configuration^15–18^. However, it is not uncommon to face cases of severe rotator cuff tears that can be repaired at the time of surgery with satisfactory outcomes, while preoperative MRIs indicate the tears to be irreparable. Also, there are some cases that appear to be reparable on preoperative images, but they are found to be barely repaired under tension or even irreparable during operation.

Although it is well known that repairing large or massive tears under tension may have an adverse effect on healing of the repaired tendons, there are only few studies addressing this issue in medium sized isolated supraspinatus full-thickness tear. Therefore, the purpose of this study was to compare the clinical outcomes and structural integrity of arthroscopically repaired medium-sized rotator cuff tears with and without tension. It was hypothesized that the repair under tension group would show inferior clinical outcomes and higher re-tear rates, compared to the repair without tension group, despite delayed rehabilitation to further consolidate tendon healing in the repair under tension group.

## Materials and methods

### Study population

**Figure 1:**
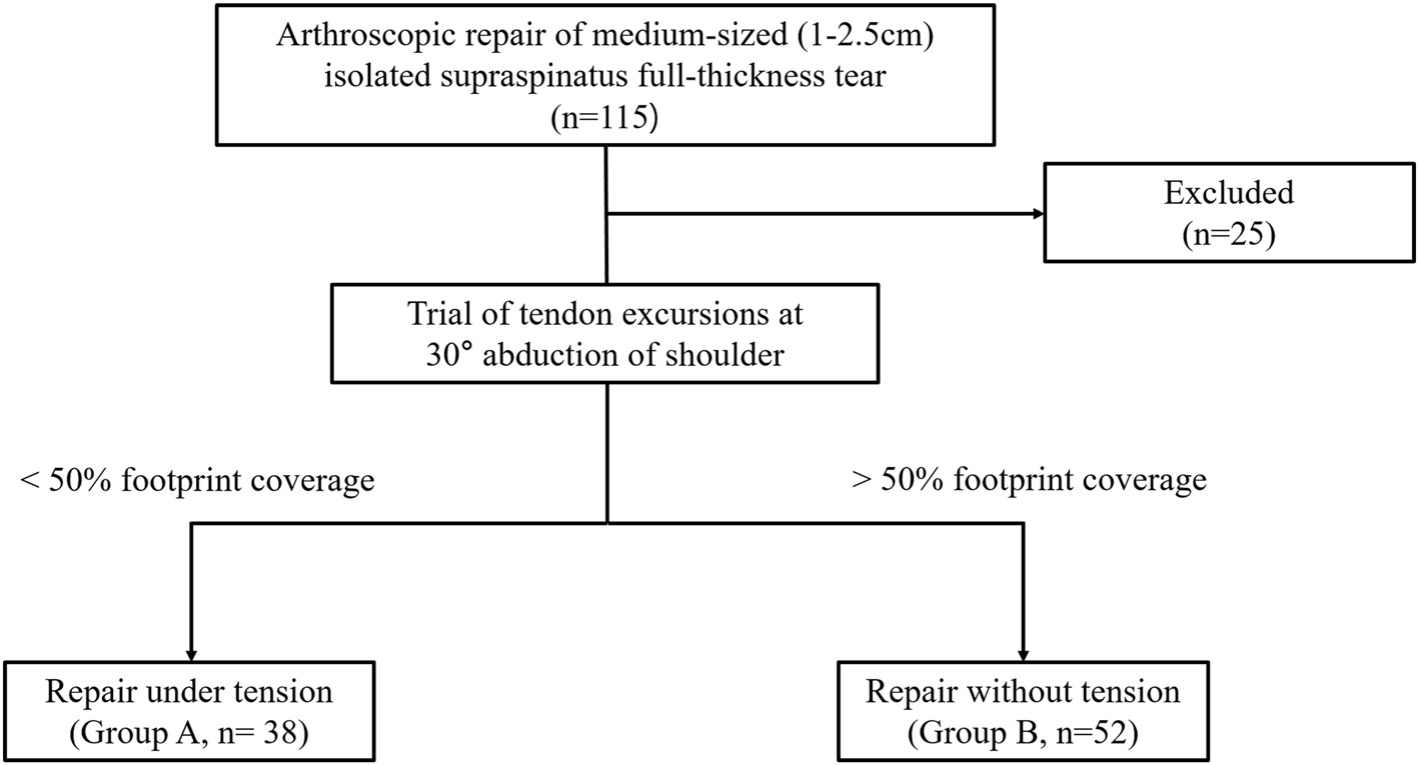
Diagram shows group assignment of this study.

This retrospective study reviewed 115 patients, who underwent arthroscopic repair of medium-sized (1–2.5 cm) isolated supraspinatus full-thickness tear from March 2014 to December 2017. These arthroscopic surgeries were performed by a single surgeon, and they were divided according to whether the repair was done under tension (Group A) or without tension (Group B). Repair under tension was defined as < 50% incomplete footprint coverage by the retracted supraspinatus tendon in 30° abduction of shoulder (Fig. 1). Indication of surgery was pain and discomfort in activities of daily living that is refractory to conservative treatments for at least 3 months. The inclusion criteria were arthroscopic repair of medium-sized (1–2.5 cm) isolated full-thickness tear in a single-row fashion and being available for a 2 years of follow-up. The exclusion criteria were (1) previous surgical history of the affected shoulder, (2) revision surgery, (3) partial repair, (4) subscapularis tear that also requires repair, (5) lack of follow-up magnetic resonance arthrography (MRA) or computed tomographic arthrography (CTA) images at postoperative 6 months, and (6) worker’s compensation claim. Finally, 90 patients (38 in Group A and 52 in Group B), who satisfied the abovementioned criteria, were included in the current study. All of the methods were performed in accordance with the International Council of Harmonization-Good Clinical Practice guidelines and the applicable laws and regulations. All patients provided their written informed consent prior to arthroscopic rotator cuff repair and the publication of their individual data. The institutional review board approved the study, and the requirement for informed consent of patients was waived (Severance Hospital IRB No. 4–2020-0335).

### Functional and radiological assessments

As functional indices, the following were evaluated: visual analog scale (VAS) for pain, subjective shoulder value (SSV), American Shoulder and Elbow Surgeon (ASES) shoulder score, and University of California Los Angeles (UCLA) shoulder score. The shoulder’s active range of motion (ROM) was assessed by three movements: forward flexion in the scapular plane, external rotation of the elbow at the side, and internal rotation. Internal rotation was examined by measuring how high the patients could reach along the spine with their thumb. The spinal segments were assigned numerical equivalents: T1-T12 as 1–12; L1-L5 as 13–17, and sacrum as 18^19–21^. During their visits, an independent examiner, who was blinded to group assignment of each patient, evaluated the initial and subsequent scores and ROMs. Preoperative radiologic assessments included standing true anteroposterior (AP) views of the shoulder in neutral and axillary positions and baseline magnetic resonance imaging (MRI) or MRA studies. To assess the structural integrity of the repaired tendons, a postoperative MRA (3.0-T MR imaging unit, MAGNETOM Tim Trio; Siemens, Erlangen, Germany) or CTA (SOMATOM Sensation 64; Siemens) was obtained 6 months after the operation.

### Surgical procedures

The patients underwent arthroscopic repair in the beach chair position under general anesthesia. Through a standard posterior portal, the joint was inspected to identify the subscapularis tendon tear or any other intra-articular lesions. In the subacromial space, excessive bursal tissue was debrided for better visualization. Viewed from the lateral portal in the subacromial space, tear configuration and mobility were checked. If necessary, the coracohumeral ligament was released (anterior interval slide). Then, potential coverage area of the footprint was estimated by mobilizing and pulling the torn and retracted supraspinatus tendon onto its original insertion site. When coverage of the footprint was less than 50%, suture anchors were placed as medially as possible within the medial half of the footprint to reduce tension after the repair. For Group B, where coverage of the footprint was more than 50%, the anchors were inserted on a such position to cover the maximum area of the footprint without tension. A suture passer was introduced through the anterolateral portal, and simple repair was completed in a single-row fashion.

### Postoperative rehabilitation

For the repair under tension group (Group A), the operated arm was absolutely immobilized in an abduction brace for 6 weeks after surgery without any exercise. After 6 weeks, self-assisted ROM exercises, such as forward flexion of the shoulder in supine position and table sliding/stretching exercises were encouraged after hot tub baths or showers. After 8 weeks, active-assisted ROM exercises commenced. Isotonic strengthening exercises with an elastic band were initiated 3 months after the operation. At postoperative 6 months, the patients were allowed to gradually resume their premorbid levels of sports activities. For the repair without tension group (Group B), self-assisted circumduction exercises were initiated and encouraged on the day after surgery, and they were educated with the same rehabilitation protocol of Group A thereafter.

### Statistical analysis

The SPSS program (IBM SPSS statistics version 25.0, IBM Corp., Armonk, NY, USA) was used for statistical analyses. The student’s t-test was used to compare continuous or continuous ranked data, such as the ROM and shoulder functional scores. The Fisher’s exact test was used to compare categorical data between the two groups, such as the presence of re-tear on follow-up MRA or CTA. Statistical significance was set at p < 0.05. For sample size calculation, ASES score was chosen as a primary variable for the calculation which was done in accordance with formerly known results on the minimal clinically important difference of the score^22^. A power calculation with an effect size of 17, a confidence level of 95% α = 0.05, and a power of 90% revealed that a minimum of 22 patients per group was required for the study.

## Results

### Patient demographics

There were 16 male and 22 female patients in Group A, and their mean age was 62.3 ± 7.4 years (range; 45–75 year-old). For Group B, there were 21 males and 31 females, and their mean age was 61.7 ± 6.1 years (range; 49–72 year-old). Preoperative symptom duration was 24.6 ± 7.8 months (range; 6–38 month) for Group A and 22.9 ± 9.1 months (range; 8–49 month) for Group B. When these demographic data were compared between the two groups, no statistical difference was found. The evaluation of preoperative fatty infiltration showed that stages II, III, and IV were eight (21.1%), 27 (71.1%), and three (7.9%) for Group A, respectively, and for Group B, there were three (5.8%) in stage 0, 11 (21.2%) in stage I, 34 (65.4%) in stage II, and four (7.7%) in stage III. The difference in fatty infiltration was statistically significant between the two groups. (p < 0.001) The mean anteroposterior dimensions of the tears measured intraoperatively were determined to be 2.2 ± 0.4 cm for Group A and 2.1 ± 0.5 cm for Group B, and comparison of the means showed no statistical significance. However, when the extent of retraction was evaluated in both groups, the mean retractions were 3.1 ± 0.4 cm for Group A and 1.8 ± 0.5 cm for Group B, showed statistically significant difference between the two (p < 0.001) (Table 1).

**Table 1:** Patient demographics of both groups.

	Group A (n = 38)	Group B (n = 52)	p value
Age (years)	62.3 ± 7.4 (range 45–75)	61.7 ± 6.1 (range 49–72)	n.s
Sex (male/female)	16/22	21/31	n.s
Symptom duration (months)	24.6 ± 7.8 (range 6–38)	22.9 ± 9.1 (range 8–49)	n.s
Grades of fatty degeneration in the supraspinatus	2.9 ± 0.4 (G2:8 G3:27 G4:3)	2.2 ± 0.9 (G0:3/ G1:11/G2:34/G3:4)	< 0.001
Tear size			
Anteroposterior dimension (cm)	2.2 ± 0.4 (range 1.3–2.5)	2.1 ± 0.5 (range 1.0–2.5)	n.s
Amount of retraction (cm)	3.1 ± 0.4 (range 2.2–3.4)	1.8 ± 0.5 (range 1.3–2.5)	< 0.001

Values are presented as means and standard deviations.

Fatty infiltration was measured according to the Goutallier classification.

### Functional and radiological outcomes

The mean VAS scores changed from the preoperative score of 6.6 ± 1.4 (range; 5–10) to 1.8 ± 1.3 (range; 0–4) at postoperative 2 years in Group A, and from 6.5 ± 1.5 (range; 4–10) initially to 1.4 ± 1.2 (range; 0–5) at postoperative 2 years in Group B. Although the scores showed significant improvement in both groups, the comparison of these postoperatively improved scores showed no significant difference between the two groups. The mean SSV scores also had considerable enhancement from the preoperative value of 31.8 ± 9.4 (range; 10–50) to the postoperative value of 89.1 ± 7.7 (range; 70–100) for Group A and from 34.8 ± 9.8 (range; 0–50) measured preoperatively to 91.2 ± 7.5 (range; 75–100) after the surgery for Group B. However, these changes in scores at 2 years after surgery showed no statistical significance between the two groups. The mean ASES score significantly improved from the initial values of 34.1 ± 6.7 (range; 18–42) and 34.3 ± 7.4 (range; 16–45) to 88.9 ± 5.8 (range; 75–95) and 90.5 ± 5.3 (range; 77–98) postoperatively for Groups A and B, respectively, but the improvements at postoperative 2 years did not show significant difference between the two groups. There were also substantial changes in the mean UCLA scores in both Groups A and B. The mean score rose from the preoperative score of 15.6 ± 3.6 (range; 9–21) to the postoperative score of 29.9 ± 3.9 (range; 20–35) for Group A, while Group B improved from 16.2 ± 3.8 (range; 9–22) to 31.2 ± 2.9 (range; 23–35) postoperatively. However, like other scores mentioned above, the mean UCLA scores measured at 2 years after surgery revealed no significant difference between the two groups (Table 2).

**Table 2:** Visual analog scale (VAS) pain score, American Shoulder and Elbow Surgeon (ASES) score, and University of California Los Angeles (UCLA) shoulder scores for both groups.

	Group A (n = 38)	Group B (n = 52)	p value
**VAS**			
Preoperative Postoperative 2 years	6.6 ± 1.4 (range 5–10) 1.8 ± 1.3 (range 0–4)	6.5 ± 1.5 (range 4–10) 1.4 ± 1.2 (range 0–5)	n.s n.s
**SSV**			
Preoperative Postoperative 2 years	31.8 ± 9.4 (range 10–50) 89.1 ± 7.7 (range 70–100)	34.8 ± 9.8 (range 0–50) 91.2 ± 7.5 (range 75–100)	n.s n.s
**ASES**			
Preoperative Postoperative 2 years	34.1 ± 6.7 (range 18–42) 88.9 ± 5.8 (range 75–95)	34.3 ± 7.4 (range 16–45) 90.5 ± 5.3 (range 77–98)	n.s n.s
**UCLA**			
Preoperative Postoperative 2 years	15.6 ± 3.6 (range 9–21) 29.9 ± 3.9 (range 20–35)	16.2 ± 3.8 (range 9–22) 31.2 ± 2.9 (range 23–35)	n.s n.s

The values are given as means and standard deviations.

Forward flexion for Groups A and B improved from 125 ± 11° (range; 110°–145°) and 128 ± 10° (range; 120°–150°) initially to 141 ± 10° (range; 120°–160°) and 145 ± 11° (range; 120°–160°) at postoperative 2 years, respectively. External rotation with the elbow at the side changed from the preoperative values of 51 ± 13° (range; 30–65°) and 54 ± 9° (range; 45°–65°) to the postoperative angles of 56 ± 8° (range; 45°–65°) and 58 ± 9° (range; 45°–70°) for Groups A and B, respectively. Internal rotation shifted from 14 ± 2 (range; 11–17) and 13 ± 3 (range; 9–17) initially to 12 ± 2 (range; 8–16) and 11 ± 2 (range; 8–15) at two years after the surgery for Groups A and B, respectively. When these active ROMs measured preoperatively and at postoperative 2 years were compared between the two groups, no statistically significant difference was evident. However, those measured at postoperative 3 months and 6 months showed statistically lower forward flexion, external rotation and internal rotation in Group A than in Group B.

The re-tear rate found in the follow-up MRA or CTA at postoperative 6 months was 28.9% (11/38) for Group A and 9.6% (5/52) for Group B, and the comparison of rates showed that Group A’s remarkably higher re-tear rate had statistically significant difference from the re-tear rate of Group B (Table 3).

**Table 3:** Active range of motion and repair integrity for both groups.

	Group A (n = 38)	Group B (n = 52)	p value
**Forward flexion (deg)**			
Preoperative	125 ± 11 (range 110–145)	128 ± 10 (range 120–150)	n.s
Postoperative 3 months	104 ± 15 (range 70–125)	119 ± 14 (range 90–135)	< 0.001
Postoperative 6 months	128 ± 12 (range 110–145)	133 ± 10 (range 120–145)	0.022
Postoperative 12 months	138 ± 11 (range 120–160)	141 ± 9 (range 120–150)	n.s
Postoperative 24 months	141 ± 10 (range 120–160)	145 ± 11 (range 120–160)	n.s
**External rotation (deg)**			
Preoperative	51 ± 13 (range 30–65)	54 ± 9 (range 40–65)	n.s
Postoperative 3 months	37 ± 10 (range 20–55)	45 ± 8 (range 30–65)	< 0.001
Postoperative 6 months	48 ± 10 (range 30–60)	53 ± 8 (range 40–65)	0.005
Postoperative 12 months	52 ± 10 (range 40–65)	55 ± 10 (range 40–70)	n.s
Postoperative 24 months	56 ± 8 (range 45–65)	58 ± 9 (range 45–70)	n.s
**Internal rotation**^a^			
Preoperative	14 ± 2 (range 11–17)	13 ± 3 (range 9–17)	n.s
Postoperative 3 months	18 ± 1 (range 14–18)	15 ± 2 (range 12–18)	< 0.001
Postoperative 6 months	15 ± 2 (range 11–18)	13 ± 2 (range 11–17)	0.029
Postoperative 12 months	13 ± 2 (range 10–17)	13 ± 2 (range 9–17)	n.s
Postoperative 24 months	12 ± 2 (range 8–16)	11 ± 2 (range 8–15)	n.s
**Re-tear rate**			
Overall, n (%)	11 (28.9)	5 (9.6)	0.018

Values are presented as means and standard deviations.

^a^Internal rotation was determined by measuring the highest spinal segment reached by the patient’s thumb. To facilitate statistical analyses, the spinal segment level was converted into numbers: T1 to T12 are represented as 1 through 12, L1 to L5 are represented as 13 through 17, and the sacrum is represented as 18.

## Discussion

The purpose of this study was to compare the clinical outcomes and structural integrity between arthroscopic rotator cuff repair with and without tension. As it was hypothesized, the repair under tension group, despite 6 weeks of absolute immobilization to protect healing of the repaired tendons, showed a significantly higher re-tear rate compared to the repair without tension group. The clinical outcomes, however, did not show statistically significant difference between the two groups, which was partially inconsistent with the hypothesis.

In large to massive rotator cuff tears with retraction, repairing the cuff under tension is common, and the effect of tension in these repaired cuff has been frequently evaluated and argued^2,4,17,23,24^. However, there also exist unexpected but empirical cases of medium-sized rotator cuff tear or isolated supraspinatus tendon tear encountering incomplete footprint coverage due to excessive tension. The authors considered that these unusual cases may show different outcomes from other more general isolated supraspinatus tendon tears that experience no excessive tension during repair and are repaired with sufficient footprint coverage. As Roth et al.^25^ indicated in their systemic review of posterosuperior rotator cuff tears, a double-row trans-osseous equivalent repair may be inadequate for massive or overly contracted tears, due to the limited tendon mobility. In massive rotator cuff tears, double-row repair may be suboptimal due to the limited mobility of the severely retracted tendons, which inflicts considerable tension on the repaired construct. Therefore, when excessive tension was expected during repair even in medium-sized isolated supraspinatus tears, the authors in the current study tried to insert suture anchors in a single-row fashion, as medially as possible within the footprint. In contrast, the cases of isolated supraspinatus medium-sized tears that had been repaired without tension and had achieved more than half or complete footprint coverage were chosen as the control group for this study.

With development in instruments and repairing techniques, many solutions have been devised to deal with difficult cases, and various studies have investigated the factors that affect healing of the repaired cuff^15,16,26–28^. As Brian et al.^16^ noted in their study, older age, larger tear size, and fatty infiltration with muscle atrophy negatively affect healing potential of the repaired rotator cuff. They also argued that postoperative rehabilitation protocol is still conflicting. Interestingly, preoperative stiffness of the affected shoulder had positive effect on healing, whereas newly developed postoperative stiffness was closely related with re-tear and worse functional outcome. There are growing efforts to predict the surgical reparability of large to massive size cuff tears. Kim et al.^29^ reported that infraspinatus fatty infiltration was considerably effective in predicting reparability, while the combination of Goutallier classification < 3 for the infraspinatus muscle and Patte classification ≤ 2 for the rotator cuff muscles was the most predictive among the other combinations of variables. Kim et al.^20^ reported on a prediction system of reparability index, which is consists of four parameters; mediolateral diameter ≥ 4.2 cm, anteroposterior diameter ≥ 3.7 cm, Warner grade ≥ 3, and Goutallier grade ≥ 3. Other researchers also reported predictable variables of reparability such as tendon involvement type, chronic psuedoparalysis, and acromiohumeral distance, but these predicting factors are not absolute, as some cases can be repaired with improved surgical techniques, despite being preoperatively recognized as potentially irreparable^18,28^. Meyer et al.^30^ reported that greater supraspinatus retraction requires longer myotendinous unit lengthening, which may in turn adversely reduce the possibility of healing. Nevertheless, it is known empirically that this is not always the case. The current study appears to offer supplementary explanation that the degree of myotendinous unit lengthening may be related to repair tension rather than the extent of tendon retraction.

The reducibility and mobility of torn tendons and the quality of repaired tendons have similar concept of variables from which several studies to verified their relations with surgical outcomes. In some previous studies, tension of the repaired tendons itself was not an independent factor for predicting re-tear, but the current study confirmed that repair under tension significantly affected the re-tear rate^16,31^. This finding was consistent with the report by Kim et al.^5^. However, although they noted that repair tension was directly proportional to the tear size, the result of the current study showed an even distribution of tear sizes between groups, with two possible explanations of it. First, the samples of the current study were limited to the medium sized tear (1–2.5 cm). Secondly, this study utilized the surgical technique of interval slide to gain additional tendon mobility, while Kim et al. did not.

In fact, previous literatures have made attempts to objectively quantify tension. Kim et al.^5^ used temporary stitch on the torn end, which was connected to tensiometer, and it was pulled to the footprint. The tensiometer was set for the measurement of peak tension (N), and the measurement was considered to be repair tension. More recently, Park et al.^6^ objectively measured the actual tension of large to massive rotator cuff tears intraoperatively and evaluated its correlation to the re-tear rate. As a result, they noted substantial increase in the re-tear rate, when more than 35 N of tension is applied to the torn tendons prior to reaching the footprint. Although their proposed cut-off value of 35 N in tension is a noteworthy conclusion, varying degrees of soft tissue laxity or adhesion found in various patients cast some doubts over whether the objective measuring of tension while pulling the retracted tendons has any clinically significant implication. Moreover, since the incidence angle between suture and footprint varies from case to case, the vector of actual tension is also changed for each mathematical reason. Therefore, the authors of the current study consider and suggest that it is more practical to evaluate how reducible the torn tendon is and how much the footprint can be covered with the retract tendon in order to evaluate the amount of tension applied during repair.

This study has several limitations. First, although the authors retrospectively compared the outcomes between repair with and without tension on medium-sized rotator cuff tear, any difference in the initial fatty infiltration grade, amount of retraction, and postoperative rehabilitation protocol may have affected the final results. Thus, when interpreting our results, this should be considered. Second, group assignment was subjective in determining the degree of footprint coverage on the greater tuberosity for assignment of the patients into two groups. Third, the statistical power of this study was low due to its relatively small sample size. Lastly, this study did not reflect the difference in tear shapes regardless of the groups.

## Conclusion

The torn cuff tendons that were repaired under tension due to retraction with limited mobility had significantly higher re-tear rate, despite having immobilized for 6 weeks postoperatively. However, their clinical outcomes showed no significant difference from the outcomes of repaired tendons without tension.
